# Mesenchymal Stem Cells Induce Directional Migration of Invasive Breast Cancer Cells through TGF-β

**DOI:** 10.1038/srep16941

**Published:** 2015-11-20

**Authors:** Kathleen M. McAndrews, Daniel J. McGrail, Nithin Ravikumar, Michelle R. Dawson

**Affiliations:** 1 School of Chemical & Biomolecular Engineering, Georgia Institute of Technology, Atlanta, GA 30332, USA; 2 The Petit Institute for Bioengineering and Bioscience, Georgia Institute of Technology, Atlanta, GA 30332, USA

## Abstract

Mesenchymal stem cells (MSCs) are recruited to the tumor microenvironment and influence tumor progression; however, how MSCs induce the invasion of cancer cells is not completely understood. Here, we used a 3D coculture model to determine how MSCs affect the migration of invasive breast cancer cells. Coculture with MSCs increases the elongation, directional migration, and traction generation of breast cancer cells. MSC-induced directional migration directly correlates with traction generation and is mediated by transforming growth factor β (TGF-β) and the migratory proteins rho-associated kinase, focal adhesion kinase, and matrix metalloproteinases. Treatment with MSC conditioned media or recombinant TGF-β1 elicits a similar migration response to coculture. Taken together, this work suggests TGF-β is secreted by MSCs, leading to force-dependent directional migration of invasive breast cancer cells. These pathways may be potential targets for blocking cancer cell invasion and subsequent metastasis.

The tumor microenvironment consists of malignant cells, a network of extracellular matrix (ECM) proteins, and a variety of recruited cells. All of these components dynamically interact to influence cancer progression. These interactions are mediated by chemical signals, including cytokines, chemokines, growth factors, and matrix remodeling proteins. In addition, mechanical signals from the tumor microenvironment can have profound effects on tumor progression^1^. Drugs that minimize the crosstalk between cells in the tumor microenvironment have been proposed as potential targets for cancer prevention^2^ and treatment^3^,^4^. A number of drugs targeting different components of the microenvironment, including blood vessels, ECM, fibroblasts, and immune cells, have been developed^4^. Sibrotuzumab was developed to target fibroblast activation protein (FAP), which is involved in matrix degradation and is expressed by fibroblasts in the tumor microenvironment^5^. In addition, imatinib targets receptor tyrosine kinases critical for fibroblast function^4^.

Mesenchymal stem cells (MSCs) are recruited from the bone marrow and local adipose tissue^6^ in response to tumor-secreted soluble factors^7^,^8^. Gene expression of stromal cells is indicative of patient prognosis^9^, suggesting these recruited cells play a critical role in regulating tumor progression. MSCs promote the growth of tumors through differentiation into carcinoma-associated fibroblasts (CAFs), angiogenesis induction, and secretion of growth factors^10^. While local adipose-derived MSCs express markers characteristic of vascular stroma (NG2, CD31, αSMA), stromal cells derived from bone marrow MSCs express high levels of CAF-associated markers FAP and fibroblast specific protein (FSP), both of which are thought to be critical for invasion and metastasis^6^. MSCs can also induce the metastasis of breast tumors through secretion of soluble factors such as CCL5^11^ and by enhancing cancer stem cell properties^12^. Coculture of MSCs with breast cancer cells induces placental growth factor (PGF) expression which promotes MSC homing *in vivo* and breast cancer metastasis in a hypoxia inducible factor (HIF)-dependent manner^13^. Thus, a better understanding of how MSCs induce the invasive properties of cancer cells could provide potential therapeutic targets for metastatic cancer.

The ECM also plays a critical role in cancer progression. During breast cancer progression, fibroblast-like cells, including MSCs, deposit laminin, fibronectin^5^, and fibrillar collagen^14^, which increases cancer cell proliferation and invasion^15^. High expression of stromal fibronectin has been associated with negative prognosis in breast cancer^16^. MSCs produce tenascin C^17^, which has been implicated in breast cancer metastasis to the lung^18^ and poor patient prognosis^19^. MSCs may also play a critical role in ECM remodeling, as the coculture of MSCs with breast cancer cells causes upregulation of lysyl oxidase (LOX)^13^, a collagen crosslinker. Previous studies have demonstrated LOX-mediated collagen crosslinking promotes breast cancer progression^20^. In addition, the mechanical properties of the ECM can induce a malignant phenotype^21^, can promote tumor progression^20^, and are critical for the generation and maintenance of the CAF phenotype^22^. In order to migrate in 3D environments, cancer cells must navigate and remodel dense ECM^23^,^24^,^25^,^26^. Two major types of migration are utilized by individual cancer cells to migrate in 3D: amoeboid and mesenchymal. Amoeboid migration is characterized by rounded cells that circumnavigate ECM without the use of adhesion proteins or matrix degradation; whereas for mesenchymal migration, cells elongate, establish integrin-mediated adhesion to the ECM, degrade ECM with matrix metalloproteinases (MMPs), and contract the cell body via myosin light-chain kinase, Rho, and ROCK^27^. Previous studies have demonstrated that fibroblasts utilize Rho-mediated matrix remodeling to generate tracks to enable the invasion of cancer cells^28^. In addition, interstitial flow causes fibroblasts to reorganize collagen fibers through Rho, which promotes cancer cell invasion^29^. Fibroblasts have similar gene expression profiles^30^ and immunomodulatory properties^31^ to MSCs; thus, we hypothesized that MSCs may induce the invasion of cancer cells through similar mechanisms.

In this study, we show that coculture with MSCs causes MDA-MB-231 invasive breast cancer cells to elongate and directionally migrate. Small molecule inhibitor studies revealed MSC-induced directional migration is mediated by TGF-β, ROCK, FAK, and MMPs, but not PDGF or VEGF. Traction generation appeared to be critical for cancer cell migration, as directional migration directly correlated with bead displacement. Treatment of cancer cells with recombinant TGF-β1 elicited a strikingly similar response to MSC coculture, suggesting that TGF-β secreted in coculture activates ROCK, FAK, and MMPs to facilitate the directional migration of cancer cells. These results elucidate how MSCs induce breast cancer cell invasion and may provide therapeutic targets to prevent invasion and metastasis.

## Results

### Coculture with MSCs induces the elongation and directional migration of breast cancer cells

**Figure 1:**
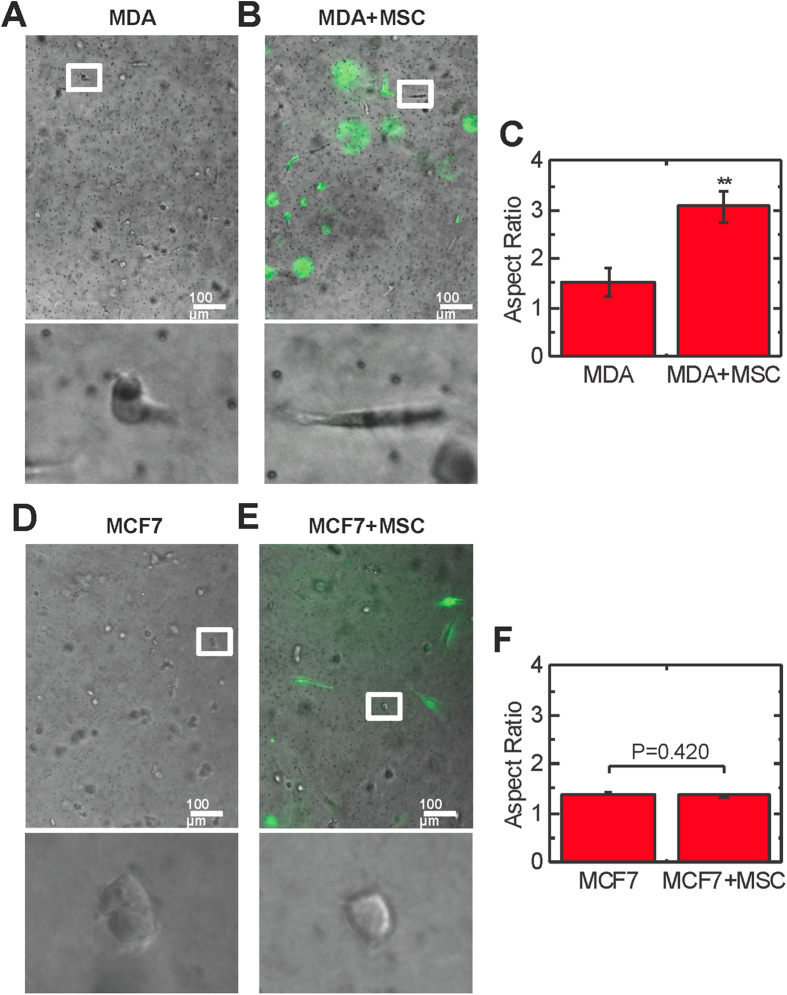
Invasive MDA cells display an elongated phenotype in coculture. Images of invasive breast cancer cells (MDA-MB-231, MDA) cultured alone (**A**) and in coculture with MSCs (**B**). MSCs are labeled with CFSE (green). Scale bar = 100 μm. (**C**) MDA cells were significantly (P < 0.01) more elongated in coculture (n = 3). Images of non-invasive breast cancer cells (MCF7) cultured alone (**D**) and in coculture with MSCs (**E**). (**F**) MCF7 cells did not elongate in coculture (P = 0.420, n = 3). Values reported as mean ± SEM. *P < 0.05, **P < 0.01, ***P < 0.001.

**Figure 2:**
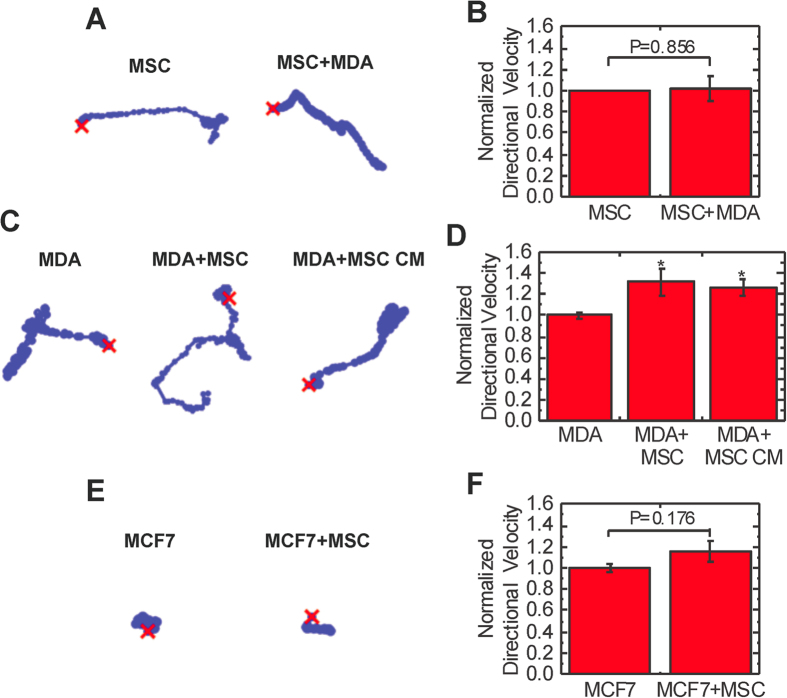
Coculture induces the migration of MDA cells but not MSCs or MCF7 cells. (**A**) Traces of MSC migration alone and in coculture. (**B**) The directional migration of MSCs alone and in coculture with MDA cells were determined by tracking cell movement over 16 hours in 3D collagen I gels (n = 7). (**C**) Traces of MDA cell migration alone, in coculture with MSCs, and with MSC CM treatment. (**D**) The directional migration of MDA cells alone, in coculture with MSCs, and with MSC CM treatment (n = 7 for MDA and MDA+MSC, n = 3 for MDA+MSC CM). (**E**) Traces of MCF7 cell migration alone and in coculture with MSCs. (**F**) The directional migration of MCF7 cells alone and in coculture with MSCs (n = 3). Statistics calulated using Kruskal-Wallis test. Values reported as mean ± SEM. *P < 0.05, **P < 0.01, *** P < 0.001.

Alterations in cell shape are critical for cell migration in 3D^32^; thus, we characterized morphological changes after coculture. MDA-MB-231 breast cancer cells (MDA) cultured alone in collagen gels remained largely unspread (Fig. 1A). Upon coculture with MSCs, MDA cells appeared more elongated (Fig. 1B). Quantification of the aspect ratio, the ratio of the major to minor axis, of cells revealed MDA cells were significantly more elongated in coculture (Fig. 1C). In contrast, the noninvasive breast cancer cell line MCF7 did not elongate in coculture with MSCs (Fig. 1D–F). Cell elongation has been associated with enhanced tumor cell invasion and metastasis^27^; consequently, we next evaluated cell migration of MSCs and MDA cells embedded in 3D collagen gels in the absence of any external stimuli or chemoattractant gradients (Fig. 2A). The migration of cells dispersed in gels was quantified over a 16 hour period. MSCs moved in a directional manner, but the presence of MDA cells did not increase their directional migration (P = 0.856, Fig. 2B). The migration of MDA cells was more random, which corresponded to a lower directional velocity than MSCs (Fig. 2B,D). Coculture with MSCs led to an increase in MDA cell directional velocity which is critical for cancer invasion^33^ (P = 0.030, Fig. 2B) as well as random migration (P = 0.026, Fig. S1A). Treatment with MSC conditioned media (MSC CM) also increased the directional migration of MDA cells (P = 0.030, Fig. 2B), indicating MSCs secrete soluble factors that induce migration. MSC coculture also increased the straightness motility parameter, a measure of persistence (P = 0.017, Fig. S1B), indicating that increased directional velocity was primarily due to increased persistence not overall motility. MSCs did not induce a significant increase in directional migration of MCF7 cells (P = 0.176, Fig. 2C). Together, these data indicate the MSC-induced migration observed is specific to invasive MDA cells.

### MSC-induced directional migration is mediated through TGFβR and mechanosensitive pathways

**Figure 3:**
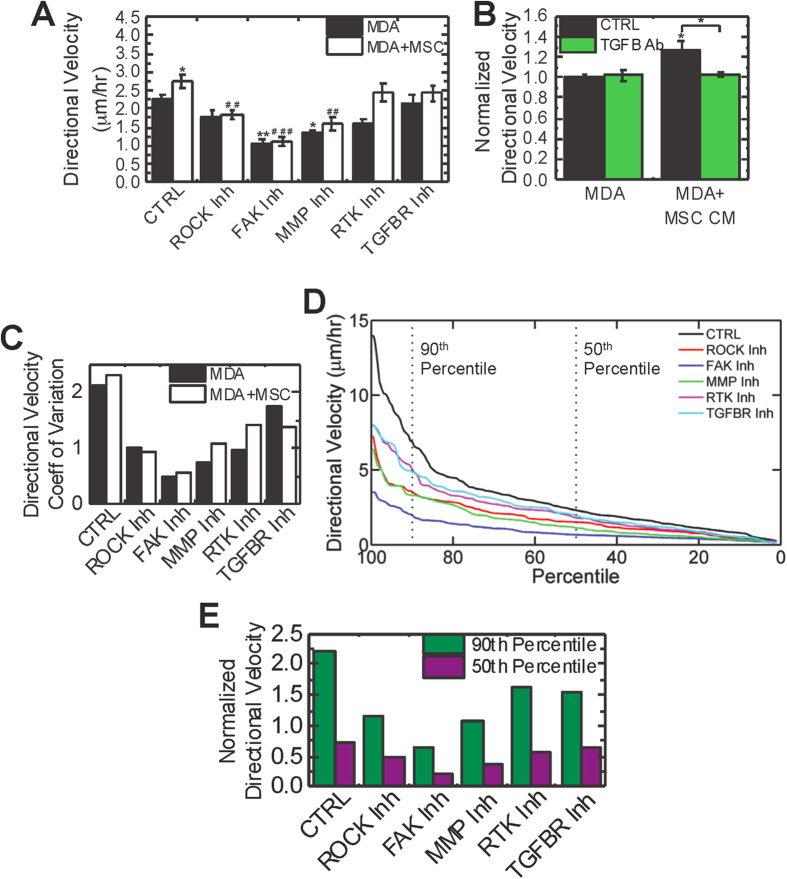
MSCs induce the directional migration of invasive breast cancer cells through TGFβR and downstream mechanotransduction pathways. (**A**) MDA cells were treated with control media (CTRL, SF DMEM), ROCK inhibitor (1 μM H-1152), FAK inhibitor (20 μM PF-573228), MMP inhibitor (20 μM GM-6001), RTK inhibitor (1 μM Sunitinib) or TGFβR inhibitor (1 μM SB-505124) 2 hours before imaging and directional velocity determined over a 16 hour period (n = 7 for CTRL, n = 4 for inhibitors). (**B**) Directional migration of MDA cells treated with control media (serum free DMEM, MDA) or MSC CM without TGF-β depletion (CTRL) or with TGF-β depletion using 10 μg/mL TGF-β1 antibody (TGFB Ab). (**C**) Directional velocity coefficient of variation (standard deviation divided by the mean) was calculated based on at least 150 individual cells for each condition. (**D**) Percentile curves of the directional velocities of at least 150 MDA cells in coculture with MSCs for each condition. Dashed lines indicate 90^th^ and 50^th^ percentile. (**E**) Directional velocity was normalized to the mean directional velocity control cells to determine relative changes in the most motile cells versus average cells. Statistics calculated using ANOVA with a Fisher LSD post-hoc test. Values reported as mean ± SEM. Significance is indicated relative to MDA control cells with *’s and relative to MDA+MSC with #’s. *P < 0.05, **P < 0.01, ***P < 0.001.

Cell elongation has been associated with the mesenchymal mode of migration, where cells utilize cell contractility, focal contacts, and MMPs to migrate^27^; thus, we hypothesized these pathways may be involved in MSC-induced directional migration of MDA cells. We targeted cell contractility with a ROCK inhibitor (H-1152), adhesion turnover with a FAK inhibitor (PF-573228), and MMP activity with a MMP inhibitor (GM-6001). Inhibition of these proteins decreased the directional velocity of MDA cells cultured alone (Fig. 3A). In addition, differences in directional migration between MDA cells alone and MDA cells in coculture with MSCs were abrogated with inhibition of ROCK, FAK, and MMPs (Fig. 3A), suggesting these pathways are critical for MSC-induced migration. In order to determine the signal upstream of these pathways, we targeted growth factor receptors known to be associated with breast cancer prognosis and metastasis, VEGFR, PDGFR and TGFβR^34^,^35^,^36^. Inhibition of VEGFR and PDGFR with a receptor tyrosine kinase (RTK) inhibitor (Sunitinib) decreased the directional velocity of MDA cells alone; however, it failed to reduce the MSC-induced directional migration response (P = 0.401). In contrast, TGFβR did not alter MDA cell directional migration when cultured alone (P = 0.303), but inhibition of this pathway abrogated differences in migration between MDA cells alone and in coculture with MSCs (Fig. 3A). These results suggest that while VEGFR and PDGFR are important for cancer cell migration, they do not mediate this MSC-induced directional migration. In order to evaluate if MSC-induced directional migration is mediated by TGF-β secreted by MSCs, we treated MDA cells with MSC CM and MSC CM depleted of TGF-β via a blocking antibody (TGFB Ab). Depletion of TGF-β from MSC CM significantly reduced (P = 0.024) directional velocity down to levels similar to MDA cells not exposed to MSC CM (Fig. 3B), indicating that TGF-β secreted by MSCs induces MDA cell directional migration.

### Inhibitors primarily target most motile cells

Heterogeneity in tumor cells has been well documented in breast cancer^37^; thus, we looked at heterogeneity in the directional velocity of breast cancer cells. Both MDA cells alone and in coculture with MSCs had a high coefficient of variation, indicating that their migration varied widely, similar to the heterogeneous gene expression observed in breast tumors^37^. We also used the coefficient of variation in directional velocity to determine if MDA cells were responding heterogeneously to inhibition of pathways critical for MSC-induced migration. Treatment with inhibitors generated a more homogenous distribution of velocities (Fig. 3C). ROCK, FAK, and MMP inhibition were associated with the lowest coefficients of variation. In coculture, MDA cells treated with both MMP and RTK inhibitors had a higher degree of heterogeneity in directional velocity compared to MDA cells cultured alone. The opposite trend was observed with TGFβR inhibitor treated cells, suggesting this inhibitor elicits a more homogenous response in coculture where TGFβR is more critical for migration. To further determine what was leading to this observed heterogeneous response, we sorted the directional velocities my magnitude and plotted their values as percentile curves of directional velocities for MDA cells in coculture with MSCs. The percentile curve was steep for MDA control cells indicating a more heterogeneous distribution of velocities, but after molecular inhibition, the curves were more shallow indicating reduced variation in directional velocity (Fig. 3D). Next, we compared the directional velocity of the 90^th^ and 50^th^ percentiles normalized to the control values for these percentiles; this is a measure of the inhibitor response in the fastest and the median moving cells (Fig. 3E). ROCK, RTK and TGFβR inhibition elicited similar responses, with the top 10% most motile cells displaying the larger differences from non-treated cells compared to average moving cells (Fig. 3E). This suggests that these inhibitors primarily target the most motile cells, with much smaller effects on less motile cells, whereas other inhibitors target all cells to a similar extent. FAK and MMP inhibitors had large effects on both the most motile cells and average moving cells; however, the decrease in directional velocity compared to control cells was similar for each of these percentiles. This indicates that in contrast to ROCK, RTK and TGFβR inhibition, FAK and MMP inhibition more equally targets all cells.

### MSCs induce cancer cell traction generation which is critical for directional migration

**Figure 4:**
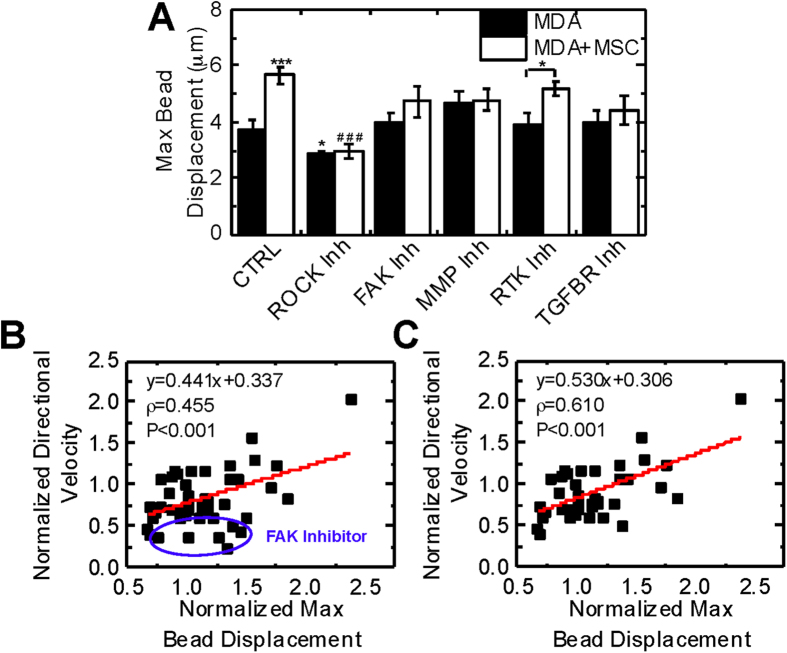
MSCs induce MDA cell traction generation which is critical for directional migration. (**A**) MDA cells were treated with control media (CTRL, SF DMEM), ROCK inhibitor (1 μM H-1152), FAK inhibitor (20 μM PF-573228), MMP inhibitor (20 μM GM-6001), RTK inhibitor (1 μM Sunitinib) or TGFβR inhibitor (1 μM SB-505124) 2 hours before imaging. 3 μm beads were embedded in the collagen gel and displacements measured over 16 hours. The maximum displacement was determined for each bead, and then max bead displacement was taken as the top 95^th^ percentile of displacements around each cell (n = 7 for CTRL, n = 4 for inhibitors). (**B**) Directional velocity correlates with max bead displacement (ρ = 0.455, P < 0.001, n = 7 for CTRL, n = 4 for inhibitors). (**C**) Cells treated with FAK inhibitor (identified with blue circle) were identified as outliers (P < 0.05) and excluded from analysis, which generated a stronger correlation between directional velocity and max bead displacement (ρ = 0.610, P < 0.001, n = 7 for CTRL, n = 4 for inhibitors). Statistics calculated using ANOVA with a Fisher LSD post-hoc test. Values reported as mean ± SEM. Significance is indicated relative to MDA control cells with *’s and relative to MDA+MSC with #’s. *P < 0.05, **P < 0.01, ***P < 0.001.

Traction generation has been implicated in tumor progression^21^ and cell motility^38^ and is critical for the mesenchymal mode of migration^27^. We measured the displacement of beads as an indicator of traction generation embedded in the collagen gel while cells were migrating. Coculture with MSCs increased the displacement of beads, and inhibition of ROCK, FAK, or MMPs abrogated this increase (Fig. 4A). ROCK inhibition, but not FAK or MMP inhibition, was associated with decreased traction generation compared to non-treated MDA cells cultured alone. In addition, RTK inhibition did not significantly alter traction generation (P = 0.416, Fig. 4B) or MSC-induced migration (Fig. 3A). Inhibition of TGFβR did not significantly alter traction for MDA cells cultured alone (P = 0.389); however, it did lead to similar traction generation alone and in coculture. These results appeared to follow trends seen with directional velocity (Fig. 3), suggesting that traction generation may be required for directional migration. We performed correlational analysis and found directional velocity correlated with bead displacement across all experimental conditions (slope = 0.441, ρ = 0.455, P < 0.001, Fig. 4C). We identified outliers in the correlation using studentized deleted residuals and observed that all outliers were cells treated with FAK inhibitor. This suggests that while active FAK is required for adhesion turnover necessary for directional migration, it is not integral for traction generation. After removal of FAK-treated cells from analysis, a stronger correlation between directional velocity and max bead displacement was observed (ρ = 0.610, P < 0.001, Fig. 4D).

### MSC secreted TGF-β induces activation of ROCK, FAK, and MMPs

**Figure 5:**
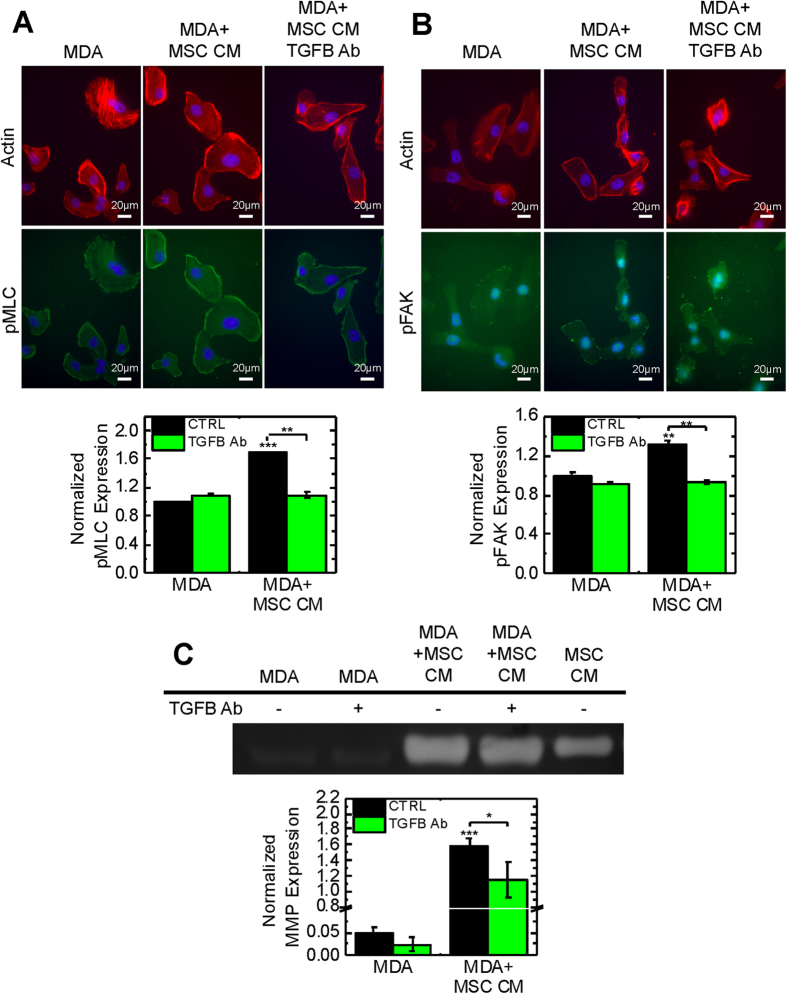
MSCs induce activation of ROCK, FAK, and MMPs in MDA cells through TGF-β secretion. MDA cells were cultured on glass coverslips and treated for 24 hours with control media (serum free DMEM, MDA) or MSC conditioned media (MSC CM) without TGF-β depletion (CTRL) or with TGF-β depletion using 10 μg/mL TGF-β1 antibody (TGFB Ab). (**A**) Immunofluorescent images of MDA cells stained for actin (red), pMLC (green), and nuclei (blue). Scale bar = 20 μm. The average intensity of pMLC was evaluated within each cell boundary determined by segmented actin images and normalized to MDA CTRL condition (n = 3). TGF-β depletion from MSC CM decreases pMLC. (**B**) Immunofluorescent images of MDA cells stained for actin (red), pFAK (green), and nuclei (blue). Scale bar = 20 μm. The average intensity of pFAK was evaluated within each cell boundary determined by segmented actin images and normalized to MDA CTRL condition (n = 3). TGF-β depletion from MSC CM decreases pFAK. For zymography experiments, MDA cells were cultured in 3D collagen gels and treated for 16 hours with control media (MDA) or MSC CM without TGF-β depletion or with TGF-β depletion. (**C**) MMP2 activity was increased in MDA cells treated with MSC CM and TGF-β depletion reverses this response. MMP activity was quantified using gelatin zymography normalized to MSC CM (n = 3). Statistics calculated using Kruskal-Wallis test. Values reported as mean ± SEM. *P < 0.05, **P < 0.01, ***P < 0.001.

Inhibitor experiments suggested TGF-β, ROCK, FAK, and MMPs were involved in MSC-induced directional migration (Fig. 3); thus, we determined if these proteins were activated in response to MSC-secreted factors. ROCK activation induces the phosphorylation of myosin light chain (pMLC) at Ser-19^39^. Immunocytochemistry for pMLC at this site was used as an indicator of ROCK activity. MDA cells increase pMLC expression in response to MSC CM and this response is abrogated by depleting TGF-β from MSC CM (Fig. 5A). In order to evaluate FAK activity, we stained for phosphorylation of FAK at Y397 (pFAK), which is inhibited by PF-573228 and is critical for cell migration^40^. The expression of pFAK is increased in a TGF-β dependent manner in MDA cells exposed to MSC CM (Fig. 5B). Gelatin zymography revealed MDA cells cultured in 3D with MSC CM increased expression of active MMP2, which was reversed with TGF-β depletion (Fig. 5C). Active MMP9 was detected; however, active MMP9 expression was very low in comparison to MMP2 (Fig. S2). Similar results were seen for MDA cells cultured on 2D coverslips (Fig. S3), indicating this response is conserved in 2D. Together, these results indicate MSCs secrete TGF-β, which is critical for ROCK, FAK, and MMP activation.

### *TGF*-β *treatment induces directional migration similar to MSC coculture*

**Figure 6:**
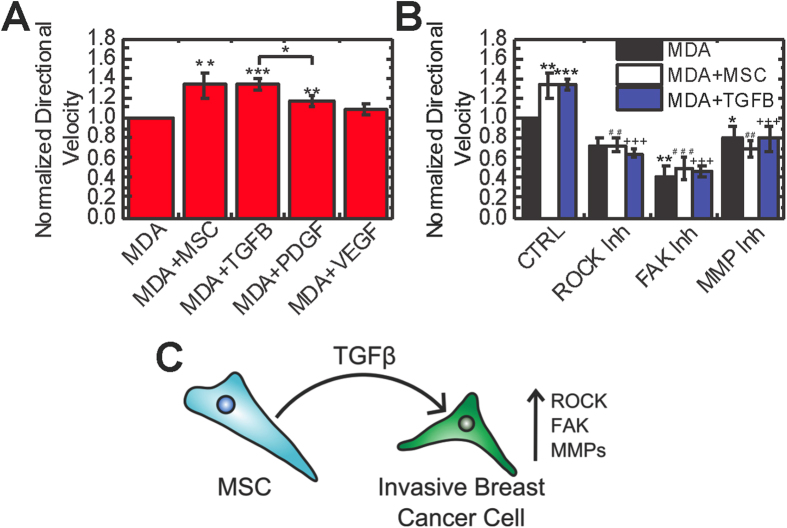
TGFβ treatment induces directional migration similar to coculture. (**A**) MDA cells were cocultured with MSCs or treated with 10 ng/mL TGF-β1, 10 ng/mL PDGF-BB, or 100 ng/mL VEGF-165 2 hours prior to imaging. Directional velocity was determined over a 16 hour period (n = 7 for MDA and MDA+MSC, n = 3 for recombinant protein treatment). (**B**) For inhibitor experiments, cells were cocultured or treated with 10 ng/mL TGF-β1 in conjunction with control media (CTRL, SF DMEM), ROCK inhibitor (1 μM H-1152), FAK inhibitor (20 μM PF-573228), MMP inhibitor (20 μM GM-6001) 2 hours prior to imaging (n = 7 for MDA and MDA+MSC, n = 4 for MDA/MDA+MSC with inhibitors, n = 3 for TGF-β1 treated cells). Directional velocity was determined over a 16 hour period. (**C**) Schematic of proposed mechanism of MSC-induced directional migration of breast cancer cells, where TGF-β is secreted in coculture which leads to activation of ROCK, FAK, and MMPs. Statistics calculated using ANOVA with a Fisher LSD post-hoc test. Values reported as mean ± SEM. *P < 0.05, **P < 0.01, ***P < 0.001.

In order to verify that TGF-β1 was the primary factor leading to increased directional migration in coculture, we treated with growth factors known to be secreted by MSCs^41^ that signal through TGFβR and RTKs to test if they induce a similar response. Treatment with TGF-β1 elicited a similar migration response to MSC coculture (Fig. 6A). To further verify these findings, we also treated with recombinant PDGF-BB and VEGF-165, the ligands for the primary receptors targeted by the RTK inhibitor Sunitinib. PDGF-BB treatment induced directional migration; however, it did not increase migration to the degree TGF-β1 or coculture did. VEGF did not induce migration of MDA cells (P = 0.404). These data suggest that the TGF-β pathway is primarily responsible for the migration observed in coculture. We then inhibited mechanotransduction pathways to determine if TGF-β was signaling through ROCK, FAK, and MMPs. Treatment with TGF-β1 in combination with these inhibitors elicited responses similar to coculture (Fig. 6B). Together these data suggest that MSCs secrete TGF-β1 which acts through ROCK, FAK, and MMPs to induce the directional migration of MDA cells (Fig. 6C).

## Discussion

TGF-β signaling is critical for directional migration (Fig. 3A); however, inhibition of TGFβR had negligible effects on random motility (Fig. S1A). Directional invasion through ECM is thought to be a critical step for breast cancer metastasis^33^, suggesting MSCs may contribute to metastasis by increasing directional migration. Blockade of ROCK and FAK abolished increased random motility in coculture, suggesting these pathways are critical for both directional and random migration. Both PDGFR decreased random motility, but there was still a significant increase in velocity in coculture. Mean velocity had a weaker correlation with traction generation (ρ = 0.330, P = 0.09) than directional velocity (Fig. S1C), indicating TGFβR is primarily involved in traction-dependent directional but not random motility. TGFβR inhibition had little effect on overall motility (mean velocity) in coculture (Fig. S1B). MMP-inhibited MDA cells showed a similar response, suggesting that activation of MMPs by TGF-β is critical to degrade the matrix for directional migration, but MMPs have little effect on random migration (mean velocity). In addition, inhibition of ROCK, FAK, and MMPs appeared to have a more potent effect on directional migration than TGFβR inhibition (Fig. 3A). MDA cells have active ROCK, FAK, and MMPs without any growth factor stimulus (Fig. 5), indicating cells maintain basal levels of these activated proteins. Thus, inhibitors of these pathways likely reduce activated protein expression to below basal levels, leading to a greater reduction in migration compared to TGFβ inhibition which reduces expression to basal levels (Fig. 5).

Active FAK is required for MSC-induced directional migration (Fig. 3A) and increased traction generation (Fig. 4A). Previous studies have demonstrated that depletion of FAK hinders migration and traction generation in 3D environments^38^; however, in 2D FAK depletion and FAK inhibition elicit different force responses^42^. Phosphorylation of the Y397 site of FAK, which is targeted by PF-573228, is critical for traction generation^43^. This site is phosphorylated by TGF-β^44^ and has been shown to be critical for growth factor-stimulated migration^45^. Treatment with TGF-β in conjunction with FAK inhibition elicited a similar migration response to coculture (Fig. 6B), suggesting that TGF-β is secreted in coculture which leads to FAK-mediated migration. The expression of pFAK was increased in MDA cells treated with MSC CM and was reversed with TGF-β depletion (Fig. 5B). FAK inhibitors have been proposed as a way to target cancer stem cells and alter chemoresistance, angiogenesis, inflammation, and profibrotic signals^40^. Our findings suggest that FAK inhibitors may also target MSC-induced directional migration.

FAK inhibitor-treated cells were outliers in the correlation between directional velocity and traction generation (Fig. 4B). Bead displacements exerted by cells treated with FAK inhibitor were high (Fig. 4A) compared to the low directional velocities of these cells (Fig. 3A) indicating the decreased velocity observed after FAK inhibition is not entirely due to decreased traction generation. Focal adhesion formation and turnover is mediated by FAK^40^, which are critical for migration in 3D^27^. Although MDA cells can still generate force after treatment with a FAK inhibitor (Fig. 4A), focal adhesion dynamics are blocked leading to inhibited migration (Fig. 3A). FAK activation also increases the expression of MMP2^46^, which in addition to cleaving ECM to facilitate migration can proteolytically activate TGF-β in the ECM^47^. Traction generated by MDA cells treated with FAK and MMP inhibitors were similar (Fig. 4A), suggesting that these two molecules may act in conjunction to promote the activation of TGF-β in the ECM. In addition, MDA cells treated with MSC CM increased MMP2 activity in a TGF-β dependent manner (Fig. 5C), indicating MSC-secreted TGF-β increases MMP2 activity. Similar migratory responses were observed with coculture and TGF-β1 treatment, indicating that MSC secreted TGF-β may activate FAK and MMPs to facilitate migration. Blockade of TGFβR in coculture inhibited migration (Fig. 3A), further supporting the hypothesis that active TGF-β signaling is required for increased directional migration of cancer cells.

Mesenchymal cells primarily utilize adhesions and cell contractility, which is mediated by Rho and ROCK, to migrate^27^. Rho also regulates actin organization, which is critical for 3D migration^27^. MDA cells display increased pMLC, an indicator of ROCK activity^39^, after exposure to MSC secreted factors (Fig. 5A). TGF-β depletion reverses this response, indicating MSC-secreted TGF-β is important for ROCK activation. ROCK inhibition acts to decrease both directional migration and traction generation in MDA cells (Figs 3A and 4A). Previous work has shown MDA cells require Rho-mediated contractility to invade into Matrigel^48^. Rho has also been implicated in the alignment of extracellular matrix fibers to facilitate invasion^49^. Our findings suggest that ROCK is also critical for MSC-induced traction generation required for the directional migration of cancer cells. Increased matrix stiffness can generate a malignant phenotype, increase traction generation, and activate Rho^21^. Coculture with MSCs was associated with higher bead displacement (Fig. 4A), suggesting that MSCs may also play a role in altering the tensional homeostasis of cancer cells, similar to ECM stiffness^21^. Previous studies have demonstrated that in 2D MSCs alter their contractile gene expression in response to tumor-secreted factors^7^,^50^ and display a myofibroblast phenotype after sustained exposure^51^. Myofibroblast contractility activates TGFβ in the ECM^52^; thus, MSC contractility may also contribute to TGF-β activation in the ECM.

Previous studies have demonstrated that PDGF activates Rac1^53^, which is required induce migration^54^. Our finding that PDGFR inhibition with Sunitinib does not alter traction generation (Fig. 4A) is in agreement with previous studies that showed Rac1 inhibition has negligible effects on traction generation in 3D^38^. PDGFR inhibition did not significantly alter directional migration and traction generation in coculture, suggesting that this pathway is not required for MSC-induced migration. Inhibition of PDGFR in MDA cells cultured alone was associated with decreased directional migration and PDGF treatment did induce modest directional migration, indicating this signal pathway does play a minor role in directional migration, but not to the same degree as TGF-β (Fig. 6A).

Inhibition of RTKs, TGFβR, and downstream migration pathways led to differential responses (Fig. 3). ROCK and FAK inhibition were associated with more homogenous distributions of directional velocity (low coefficient of variation) of MDA cells alone and in coculture, whereas MMP, RTK, and TGFβR inhibition led to more heterogeneous responses (high coefficient of variation, Fig. 3C). Cells may differentially activate these proteins, leading to a heterogeneous response to inhibitors. FAK, MMP, and RTK inhibition equally targeted the top 10% and 50% most motile cells, suggesting these pathways are critical for directional migration of all cells (Fig. 3E). Both ROCK and TGFβR appeared to be critical for the directional migration of the most motile cells, as inhibition of these pathways preferentially targeted the fastest 10% of cells.

These studies identify TGFβR as a potential target to prevent MSC-induced breast cancer cell directional migration. MSCs differentiate into carcinoma-associated fibroblasts (CAFs) in response to soluble factors secreted by tumor cells^51^. Recent studies have shown the CAF phenotype is associated with poor patient prognosis and TGF-β secreted by these cells can increase the frequency of tumor-initiating cells. By blocking TGF-β crosstalk between CAFs and cancer cells, metastasis was blocked^9^. Our studies suggest the blockade of metastasis may have been in part to decreased directional migration of cancer cells, which is thought to be critical for escape from the primary tumor site^33^. TGF-β can also directly induce epithelial to mesenchymal transition (EMT), where cells transition from an epithelial phenotype to an invasive mesenchymal phenotype allowing for escape from the primary tumor site^55^. EMT induced by TGF-β has also been implicated in the activation of stromal cells to CAFs, which further promote tumor progression^5^. This indicates that TGF-β secreted by MSCs may induce EMT in addition to acting to directly increase directional migration through mechanosensitive pathways, further promoting metastasis.

MCF7 were largely non-motile in collagen gels (Fig. 2), likely because they display an epithelial phenotype^56^. Previous studies have demonstrated that breast cancer cells treated with MSC conditioned media^57^ or TGF-β^58^ show early signs of epithelial to mesenchymal transition (EMT), which generates a more motile phenotype; however, this transition occurs after 3–7 days of exposure to these factors^57^,^58^. Although MCF7 cells display some markers of EMT, heterogeneous expression of E-cadherin and vimentin is observed^58^, suggesting that these cells have not undergone a complete transition to a mesenchymal phenotype. Other studies have indicated the chromatin structure of MCF7 cells does not allow for full EMT^59^. Together, these studies indicate that MSCs do not induce migration of MCF7 cells on the time scale probed in our motility experiments due to the epithelial phenotype of MCF7 cells. Longer coculture experiments may induce EMT and MCF7 cell migration; however, it will likely be lower than MDA cell migration.

In conclusion, we demonstrated that MSCs induce the elongation and traction-dependent directional migration of invasive MDA cells. Targeting TGF-β signaling, ROCK, FAK and MMPs abrogates directional migration and traction generation differences in coculture. These data suggest TGF-β is secreted by MSCs, which leads to the activation of ROCK, FAK, and MMPs to mediate directional migration of MDA cells. Together, this work provides insight into MSC interactions with invasive breast cancer cells within the tumor microenvironment and potential therapeutic targets to halt invasion and metastasis.

## Methods

### Cell Culture

Human MSCs (Donor 7071) were obtained from Texas A&M Institute for Regenerative Medicine and cultured in αMEM (Corning) with 20% FBS (Atlanta Biologicals), 1% penicillin-streptomycin (Corning), and 1% L-glutamine (Corning). MDA-MB-231 cells (ATCC) were cultured in low glucose DMEM (Corning) supplemented with 10% FBS and 1% penicillin-streptomycin. MCF7 cells (ATCC) were cultured in RPMI (Corning) supplemented with 10% FBS and 1% penicillin-streptomycin. MSC conditioned media (MSC CM) was collected from MSCs cultured in serum-free DMEM (Corning) for 24 hours.

### Fabrication of 3D collagen gels

Cells were embedded in collagen gels as described^38^. MSCs were labeled with carboxyfluorescein succinimidyl ester (CFSE, Biolegend) in HBSS. Cells were mixed with 10× reconstitution buffer (200 μM sodium bicarbonate and 200 μM HEPES in water) and 3 μm polystyrene particles (Polysciences) and added to rat tail collagen I to obtain a 2 mg/mL collagen gel. For coculture experiments, MSCs and MDA-MB-231 cells were mixed at a 1:1 ratio. Gels were polymerized on ice for 45 minutes followed by incubation at 37 °C for 2 hours before adding RPMI with 10% FBS and 1% penicillin-streptomycin.

### Cell migration experiments

Cells were serum starved for at least 6 hours before imaging. ROCK (1 μM H-1152, Enzo), FAK (20 μM PF-573228, Sigma), MMP (20 μM GM-6001, EMD Millipore), RTK (1 μM Sunitinib, Sigma), and TGFβR (1 μM SB-505124, Sigma) inhibitors were added 2 hours before imaging. For growth factor experiments, 10 ng/mL TGF-β1 (Biolegend), 10 ng/mL PDGF-BB (Biolegend), and 100 ng/mL VEGF-165 (Biolegend) and inhibitors were added to cells 2 hours prior to imaging. For MSC CM and TGF-β depletion experiments, control media (CM, serum free DMEM) or MSC CM was added with 10 ng/mL TGF-β1 antibody (Biolegend) 2 hours prior to imaging. Imaging was performed on a Nikon Eclipse Ti inverted epifluorescent microscope with a 10× objective. Cells were maintained at 37 °C with 5% CO_2_ using an *In Vivo* Scientific environmental cell chamber and a Bioscience Tools CO_2_ controller and imaged every 5 minutes for 16 hours using a Photometrics CoolSNAP camera. For cell shape analysis, cells were manually traced in ImageJ software (NIH). The x-y coordinates of cells were determined using Metamorph software and used to evaluate motility parameters in a custom-written MATLAB algorithm. Cells that divided during the experiment were excluded from analysis. Cell velocities were calculated over 30 minute intervals and averaged to determine mean velocity. Directional velocity was calculated as the total distance traveled divided by time. Directional velocity coefficient of variation (standard deviation divided by the mean) and percentile analyses were calculated based on at least 150 individual cells.

### Bead Displacement Quantification

Bead positions were identified as described with minor modifications and trajectories linked using a Hungarian linker algorithm^60^,^61^. In brief, bright field particle images were inverted to create a bright particle on a dark background. Next, a bandpass filter was applied to the images before determining the particle centroid to subpixel resolution based on the intensity-weighted centroid. Only beads within a 75 μm radius of a cell were used for analysis; beads outside this radius were used to assess any drift over the course of imaging. Beads were assigned to each cell using a nearest-neighbor algorithm which was verified manually for each video. The maximum displacement was determined for each bead, and then max bead displacement was taken as the top 95^th^ percentile of displacements around each cell.

### Immunostaining and Quantification

Serum-starved MDA-MB-231 cells on glass coverslips were cultured in either control media (CM, serum-free DMEM) or MSC CM with 10 ng/mL TGF-β1 antibody for 24 hours. Cells were stained as previously described for F-actin and phosphorylated myosin light chain (Ser19, pMLC, Cell Signaling Technology)^62^ or phosphorylated focal adhesion kinase (Y397, pFAK, Genetex)^63^. Images were captured using a 40× oil immersion lens on an inverted Nikon Microscope with a CoolSNAP camera (Photometrics). Cell boundaries were segmented from the F-actin stain by Otsu’s method, and then the average intensity of either pMLC or pFAK determined within cells after background subtraction. All image analysis was performed in MATLAB.

### Gelatin Zymography

Serum-starved MDA-MB-231 cells embedded in collagen gels were cultured in either control media (CM, serum-free DMEM) or MSC CM with 10 ng/mL TGF-β1 antibody for 16 hours. Supernatants were then evaluated for gelatinase activity as described with minor modifications^64^. First, supernatants were mixed with 4× sample buffer (250 nM Tris-HCl, 40% v/v glycerol, 8% w/v SDS, and 0.01% w/v bromophenol blue; pH 6.8) and incubated for 10 minutes before electrophoretic separation in a 10% polyacrylamide gel embedded with 0.1% w/v gelatin. Gels were rinsed in water before two sequential incubations with renaturing buffer (developing buffer with 1% Triton-X100) for 20 minutes at room temperature. Gels were again rinsed in water before equilibrating with developing buffer (50 mM Tris-HCl, 200 mM NaCl, 5 mM CaCl2, and 0.02% w/v Brij 35; pH 7.8) for 60 minutes. After moving to fresh developing buffer gels were incubated for 16 hours at 37 °C. Gels were then rinsed with water and stained with 0.25% w/v Coomassie diluted in 10% v/v methanol, 10% v/v acetic acid, 80% v/v water. Finally, gels were destained with 10% v/v methanol, 10% v/v acetic acid, 80% v/v water until bands became clear. Images were captured in a BioRad GelDoc and quantified in ImageJ (NIH).

### Statistics

Data are reported as the mean ± standard error of the mean (SEM) for at least 3 experiments unless otherwise noted. A student t-test was used to determine significance with P < 0.05 being statistically significant (*P < 0.05, ** P < 0.01, ***P < 0.001). For normalized data with less than 3 conditions, a Kruskal-Wallis test was used to determine significance with P < 0.05 being statistically significant (*P < 0.05, **P < 0.01, ***P < 0.001). ANOVA in conjunction with a Fisher LSD post-hoc test was used for experiments with more than 3 treatment conditions with P < 0.05 being statistically significant (*P < 0.05, **P < 0.01, ***P < 0.001). For correlation analysis, Pearson’s correlation coefficients were calculated in MATLAB, with ρ = −1 being perfectly negatively correlated and ρ = + 1 being perfectly positively correlated. Studentized deleted residuals were used to identify outliers from the data with 95% confidence.

## Additional Information

**How to cite this article**: McAndrews, K. M. *et al.* Mesenchymal Stem Cells Induce Directional Migration of Invasive Breast Cancer Cells through TGF-β. *Sci. Rep.* **5**, 16941; doi: 10.1038/srep16941 (2015).

## Supplementary Material

Supplementary Information
