# Supplementary material for: Mesenchymal Stem Cells Induce Directional Migration of Invasive Breast Cancer Cells through TGF-β
Source: Sci Rep. 2015 Nov 20;5:16941. doi: 10.1038/srep16941 (PMC4653660; doi:10.1038/srep16941)
Supplement: Supplementary Information [file srep16941-s1.pdf]

Mesenchymal Stem Cells Induce Directional Migration of Invasive Breast Cancer Cells through TGF-β

Kathleen M. McAndrews[1], Daniel J. McGrail[1], Nithin Ravikumar[1], Michelle R. Dawson[1,2*]

[1] School of Chemical & Biomolecular Engineering, Georgia Institute of Technology, Atlanta, GA 30332, USA
[2] The Petit Institute for Bioengineering and Bioscience, Georgia Institute of Technology, Atlanta, GA 30332, USA

Supplemental Figures

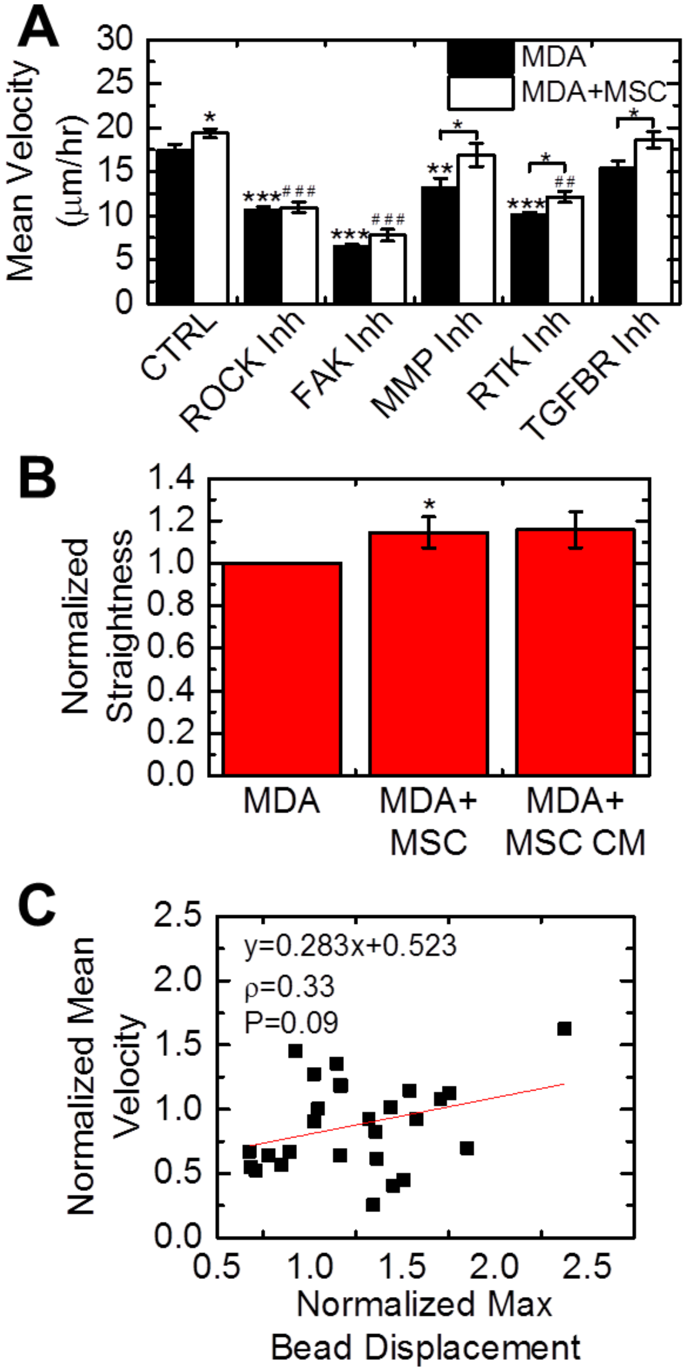

Fig S1: TGFβR is involved in directional migration but not overall motility. MDA were treated with control media (CTRL, SF DMEM), ROCK inhibitor (1 μM H-1152), FAK inhibitor (20 μM PF-573228), MMP inhibitor (20 μM GM-6001), RTK inhibitor (1 μM Sunitinib) or

TGFβR inhibitor (1 μM SB-505124). 2 hours before imaging and mean velocity determined over a 16 hour period. Mean velocity does not significantly correlate with max bead displacement ($\rho=0.33$, $P=0.09$). Statistics calculated using ANOVA with a Fisher LSD post-hoc test for mean velocity and Kruskal-Wallis test for straightness. Values reported as mean ± SEM. Significance is indicated relative to MDA control cells with *'s and relative to MDA+MSC with #'s. * $P<0.05$, ** $P<0.01$, *** $P<0.001$.

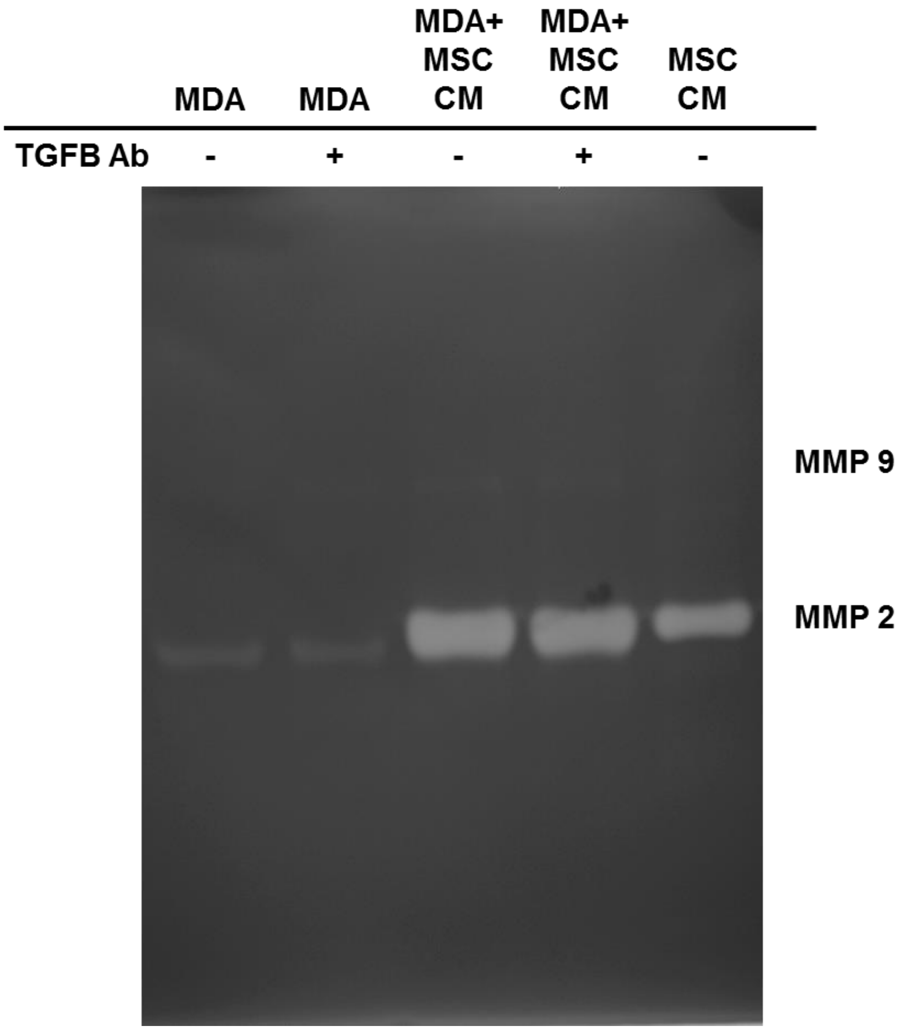

Fig S2: Full size polyacrylamide gel for gelatin zymography. MDA expressed higher levels of active MMP2 than MMP9.

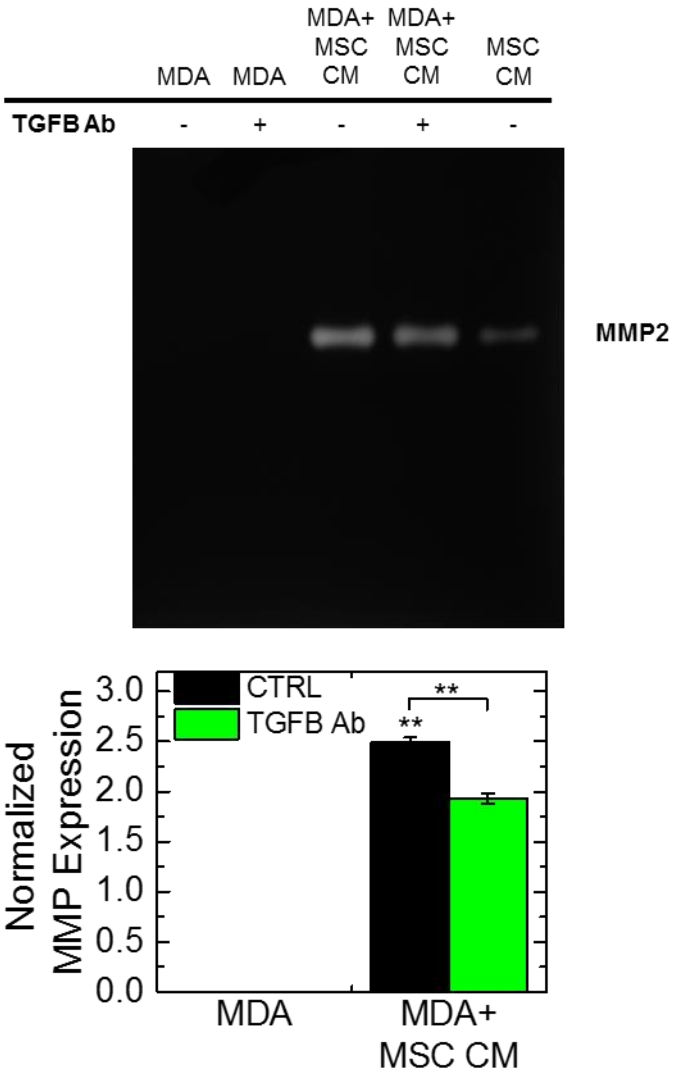

Fig. S3: MSCs induce activation of MMPs in MDA cells cultured in 2D through TGF-β secretion. MDA cells were cultured in 3D collagen gels and treated for 16 hours with control media (serum free DMEM, MDA) or MSC conditioned media (MSC CM) without TGF-β depletion (CTRL) or with TGF-β depletion using 10 μg/mL TGF-β1 antibody (TGFB Ab). MMP2 activity was increased in MDA cells cultured on glass coverslips treated with MSC CM and TGF-β depletion reverses this response. MMP activity was quantified using gelatin zymography normalized to MSC CM (n=3). Statistics calculated using Kruskal-Wallis test. Values reported as mean ± SEM. * $P<0.05$, ** $P<0.01$, *** $P<0.001$.
